# Metabolomic analysis of halotolerant endophytic bacterium *Salinivibrio costicola* isolated from *Suaeda maritima* (L.) dumort

**DOI:** 10.3389/fmolb.2022.967945

**Published:** 2022-09-02

**Authors:** Jaeyoun Lee, Soohyun Um, Seung Hyun Kim

**Affiliations:** College of Pharmacy, Yonsei Institute of Pharmaceutical Sciences, Yonsei University, Incheon, South Korea

**Keywords:** secondary metabolites, halophytic endophyte, *Salinivibrio costicola*, *Suaeda maritima* (L.) dumort., polyhydroxybuthyrate (PHB)

## Abstract

In this study, the *Salinivibrio costicola* strain was isolated from *Suaeda maritima* (L.) Dumort. collected in Sinan, Republic of Korea. The endophytic characteristics of the Gram-negative bacterium *S*. *costicola* were verified with metagenomics sequencing of *S. maritima*. *S*. *costicola* was cultivated for 3 days in a liquid medium with 3.3% sea salt and analyzed the metabolites produced by the strain cultured in five different bacterial cultivation media. From the bacterial cultures, polyhydroxybutyrate derivatives were detected using high-resolution mass spectrometry, and three major compounds were isolated by high-performance liquid chromatography. The chemical structures of the compounds were elucidated using nuclear magnetic resonance and MS analyses. The relationship between the compounds was confirmed with Global Natural Product Social Molecular Networking, which showed clustering of the compounds. From the *S. maritima* extract, polyhydroxybutyrate derivatives produced by *S. costicola* were detected as being accumulated in the host plant.

## Introduction

The close relationship between plants and their endophytes indicates that individual host plants and microbial communities are essential for thriving and survival (Kratz, 1991; Compant et al., 2010; White et al., 2019). Despite the pervasiveness of these mutualistic interactions, the molecular underpinnings of most of them either remain unexplored or are only being understood. In recent years, several studies have emphasized the importance of plant-microbe relationships as particularly attractive sources for new bioactive small molecules, many of which may have potential applications in the pharmaceutical industry (Helfrich et al., 2018; Newman, 2018). The discovery of natural products from holobiont phenotype analysis through metagenomics, the study of the collective genome of a microbial community in environmental samples, has been established over the past few years (Fuhrman, 2012; Iquebal et al., 2022; Liu et al., 2022). Metagenomic research on plant microbiomes has yielded further insights into functional information beyond the genome information of particular bacterial strains (Lucaciu et al., 2019; Liu et al., 2022). In addition, it is possible to consider both the computational and experimental aspects of molecular plant-microbiome interactions (Lucaciu et al., 2019).

This study hypothesized that the native coastal annual halophyte, *S*. *maritima* (Amaranthaceae), has habitat-specific microbial communities to overcome osmotic pressure stress, which may have evolved adaptive traits in response to high-salt environments (Clipson, 1987; Cherian and Reddy, 2000). Known to be a salt-marsh herb, it has leaves that are pale green, which changes to blackish red in autumn, and are fleshy and 1–5 cm long. The whole plant is 30–60 cm long, with branches spreading from near the ground. *S. maritima* is most common in lower marshes, where it grows in single colonies with scattered individuals (Chapman, 1947). We assumed that the microorganisms associated with *S*. *maritima* endosphere might be beneficial to the host. The purpose of this study was to investigate the assembly and structure of *in situ* bacterial microbiomes associated with the endosphere using metagenomic analysis. In addition, we isolated endophytes of *S*. *maritima* exhibiting microbial features associated with salt adaptation and estimated functional extensions of plant phenotypes within a phylogenetic framework (Yeo and Flowers, 1980; Lee et al., 2003).

## Materials and methods

### Plant collection

The plant material*, Suaeda maritima* (L.) Dumort. which is a halophyte, was collected from the Taepyeong saltern (34° 59′ 53.0″ N 126° 10′ 19.4″ E) in Sinan, South Korea in October 2021.

### DNA extraction for metagenomics

DNA preparation was performed using the FastDNA^®^ Spin Kit for soil (MP Biomedicals), and the quantity of DNA was measured with an Epoch™ Spectrometer (BioTek). Hypervariable regions V3-V4 of the bacterial 16S rRNA gene were amplified using the primer pair 341F [5′-TCGTCGGCAGCGTC-AGATGTGTATAAGAGACAG-CCTACGGGNGGCWGCAG-3' (nextera consensus - sequencing adaptor - target sequence), 50mer] and 805R [5′- GTCTCGTGGGCTCGG-AGATGTGTATAAGAGACAG-GACTACHVGGGTATCTAATCC-3' (nextera consensus - sequencing adaptor - target sequence), 55mer] containing the appropriate Illumina adapters following Illumina’s 16S Metagenomics Protocol for the first amplicon PCR. The second PCR (index PCR) was performed with forward index i5 [AATGATACGGCGACCACCGAGATCTACAC-XXXXXXXX-TCGTCGGCAGCGTC, composition: left–i5 (XXXXXXXX)–right, 51mer] and reverse index i7 [CAA​GCA​GAA​GAC​GGC​ATA​CGA​GAT-GTC​TCG​TGG​GCT​CGG, composition: left–i5 (XXXXXXXX)–right, 47mer]. PCR reagents (25 μL in total) were 10X buffer (2.5 μL), dNTP (2.5 μL), forward primer (10 pmole/μL, 1 μL), reverse primer (10 pmole/μL, 1 μL), Taq polymerase (TaKaRa Ex Taq DNA polymerase, 1000 U), DNA (2 μL), and D.W. (15.75 μL). The quantity and quality of a single library were determined using the Quanti-iT PicoGreen dsDNA Assay Kit (Invitrogen) and Agilent 2,100 Bioanalyzer System (Agilent Technologies, US.). The samples were sequenced paired-end using a MiSeq^®^ Reagent Kit v2 (500 cycles; Illumina, Inc.).

### Bioinformatics analysis

To obtain Q-values by sequence, sequences that could be misidentified as ‘new species’ by Q-value were removed. Illumina next-generation sequencing (NGS) data were analyzed using Trimmomatic software, and other NGS data were analyzed using self-developed code. The Pandaseq software was used for bioinformatics, but this was not performed for single-end reads or PacBio’s CCS data (Bartram et al., 2011). CJ Bioscience pipeline adopting in-house EzBioCloud 16S rRNA database was used with in-house algorithms to remove 16S rRNA PCR primer sequences, as these primer regions originated from the annealing process. To reduce computational cost and increase accuracy, different versions of the reference database were used to match various 16S rRNA regions. A combination of in-house algorithms and HMMER software was used for further analyses. Raw NGS data contained approximately 0.5% sequencing errors, which may occur randomly. However, as the same gene was sequenced several times during NGS experiments, it was possible to correct these errors through adequate modeling. Quality-controlled sequences were used for taxonomic assignment. The pipeline uses proven identification algorithms implemented in EzBioCloud to identify all the sequences. This two-step algorithm consists of (i) searching reference databases using the USEARCH program, and (ii) calculating similarity with robust pairwise alignment. Query sequences that matched the reference sequences in EzBioCloud by ≥ 97% similarity were considered to be at the species level. Lower sequence similarity cutoffs were used for genus or higher taxonomic levels. *x = sequence similarity to reference sequences; species (x ≥ 97%), genus (97 > x ≥ 94.5%), family (94.5 > x ≥ 86.5%), order (86.5 > x ≥ 82%), class (82 > x ≥ 78.5%), phylum (78.5 > x ≥ 75%). Cutoff values from Yarza et al. were considered for this study (Yarza et al., 2014). However, during the detection of chimeric sequences, a sequence with a similarity of ≥97% in the identification step that was previously been identified at the species level was found; therefore, it was removed from the detection target. Operational taxonomic units (OTUs) are arbitrary classification units that are often referred to as a ‘species’in microbial community analysis.

### Taxonomic analysis, alpha- and beta-diversity

All the sequences in the samples were grouped into multiple OTUs using various algorithms and programs. 1) Except for chimeras, sequences with ≥97% similarity were classified as species and each was assigned an OTU. 2) For sequences with <97% similarity, CD-hit and UCLUST programs were used to perform cluster analysis, and each cluster was assigned an OTU. 3) The OTUs from steps 1 and 2 were combined to obtain the final OTU information. 4) The OTU formed in step 2 as a single sequence was removed from the diversity analysis because it caused the diversity index to increase more than the actual value. It calculates various alpha diversity indices within a single sample using OTU information (the number of OTUs and the number of sequences in an OTU). Shannon, Simpson, and other species abundance and evenness statistics were included in alpha diversity.

### Bacteria isolation, identification, and cultivation procedures

To remove exterior contaminants, whole parts of *S*. *maritima* were disinfected by soaking in 5 percent sodium hypochlorite for 5 minutes and wiping with 80 percent aqueous ethanol. The sterile leaves, stems, and roots of *S*. *maritima* were sliced into flakes and added to a chitin medium with sea salt (6 g of chitin, 0.75 g of K_2_HPO_4_, 0.5 g of MgSO_4_.7H_2_O, and 3.5 g of K_2_HPO_4_, 10 mg of FeSO_4_.7H_2_O, 10 mg of MnCl_2_.4H_2_O, 10 mg of ZnSO_4_.7H_2_O, 100 mg of cycloheximide, 33 g of sea salt, and 36 g of agar per 1 L of sterilized water), Czapek-Dox medium with sea salt (30 g of sucrose, 2 g of NaNO_3_, 1 g of K_2_HPO_4_, 0.5 g of MgCl_2_, 0.5 g of KCl, 0.01 g of FeCl_2_, 100 mg of cycloheximide, 33 g of sea salt and 18 g of agar per 1 L of sterilized water), and A1 medium with sea salt (10 g of starch, 4 g of yeast extract, 2 g of peptone, 100 mg of cycloheximide, 33 g of sea salt, and 18 g of agar per 1 L of sterilized water) for 14 days to isolate endophytes from *S*. *maritima*. Each isolated endophyte was cultured in a modified K medium with sea salt [12 g of Luria-Bertani (LB), 12 g of potato dextrose broth (PDB), 1 g of tryptic soy broth (TSB), 33 g of sea salt, and 18 g of agar per 1 L of sterilized water at 28 °C for 6 days].

For identification of the isolated bacteria, DNA was extracted using the MagAttract HMW DNA Kit (Qiagen) according to the manufacturer’s instructions. The DNA extract was amplified with the following conditions: initial denaturation at 95°C for 5 min, followed by 30 cycles of denaturation at 95°C for 30 s, annealing at 55°C, and extension at 68°C for 1.5 min, and a final extension at 68°C for 10 min. The purified PCR products were sequenced in the forward and reverse directions in separate reactions and duplicates. The PCR amplification primer sequences were as follows: 27F (5′-AGA GTT TGA TCM TGG CTC AG-3′) and 1492R (5′-GGT TAC CTT GTT ACG ACT T-3′) for the 16S rRNA. The purified PCR products were sequenced using two primer pairs: 785F (5′-GGA TTA GAT ACC CTG GTA-3′) and 907R (5′-CCG TCA ATT CMT TTR AGT TT-3′) for 16S rRNA and NS1 (5′-GTA GTC ATA TGC TTG TCT C-3′). Sequencing was performed using a Big Dye Terminator 3.1 cycle Sequencing Kit (Applied BioSystems, United States ).

### Analyses of secondary metabolites from *S*. *costicola* YSL5

After isolating the single bacterial strains from a chitin medium with sea salt, the isolated Gram-negative bacteria (see Supplementary Figure S1), *S*. *costicola* YSL5, were cultivated in a modified K liquid medium with sea salt for 3 days (28°C, 180 rpm). Then, 10 ml of the bacterial culture was inoculated into diverse bacterial culture media, including K medium with salt (12 g of LB medium, 12 g of PDB, 1 g of TSB, 33 g of sea salt per 1 L of sterilized water), potato dextrose broth (PDB) medium with salt (27 g of potato extract and 33 g of sea salt per 1 L of sterilized water), potato dextrose yeast (PDY) media with salt (26.5 g of potato extract, 5 g of yeast extract, and 33 g of sea salt per 1 L of sterilized water), yeast extract-malt extract (YEME) medium with salt (5 g of malt extract, 2 g of yeast extract, and 33 g of sea salt per 1 L of sterilized water), and yeast peptone mannitol (YPM) medium with salt (25 g of mannitol, 5 g of yeast extract, 3 g of peptone, and 33 g of sea salt per 1 L of sterilized water) in 250 ml Erlenmeyer flask (28°C, 3 days). Each culture was extracted with ethyl acetate/water layer separation using a separatory funnel, and the ethyl acetate layer was concentrated *in vacuo*. The dried crude extract was dissolved in methanol at a concentration of 250 μg/ml and analyzed using liquid chromatography-mass spectrometry (LC-MS). High-performance liquid chromatography (HPLC) measurements were performed with an Agilent 1,290 ultra-high-performance liquid-chromatography (UHPLC) system (Agilent Technologies, Santa Clara, United States ) equipped with a 1,290 Infinity binary pump and a YMC-triart C18 column (150 × 2.0 mm, 1.9 μm; YMC KOREA Co., Seongnam, Korea). The mobile phases were 0.1% formic acid in water (A) and 0.1% formic acid in acetonitrile (B) with the following gradients: 10–100% B (0–20 min), 100% B (20.1–25 min), 100–10% B (25.1–30 min), and 10% B (30.1–35 min) with an injection in six replicates at a volume of 10 μL. Mass spectrometry was performed using an Agilent 6,530 quadrupole time-of-flight mass spectrometer (Q-TOF-MS, Agilent Technologies, Santa Clara, United States ) equipped with an electrospray ionization (ESI) source. The MS experiment was performed under the following conditions: source temperature, 100 °C; desolvation temperature, 300°C; desolvation gas flow rate, 500 L/h; cone gas flow rate, 30 L/h; capillary voltage +3.0 kV; positive and negative modes; cone voltage, 60 V; extractor voltage, 3 V.

The modified K medium was used for high-resolution tandem mass spectrometry (HRMS/MS)-based Global Natural Product Social Molecular Networking (GNPS) analysis of the crude ethyl acetate extracts obtained from 3 days cultures of *S*. *costicola* YSL5 and the methanol extract of *S*. *maritima*. Based on the tandem mass spectrometry (MS/MS) data from an Agilent 6530 Q-TOF-MS, molecular networks of the secondary metabolites were created using the GNPS platform (http://gnps.ucsd.edu) (Wang et al., 2016; Nothias et al., 2020). The data were converted to *mzML* format using MS-Convert. Using the GNPS Web server, the converted files were used to generate an MS/MS molecular network. Both the precursor ion mass tolerance and the fragment ion mass tolerance were adjusted to 2.0 Da. The default value for both was 0.5 Da. Molecular networks were generated using a cosine score of 0.7, a minimum of six matched peaks, and a minimum cluster size of 2. After analysis, data were visualized using Cytoscape 3.9.0 software (Shannon et al., 2003).

With *mzML* files of six replicates of each medium, mass feature detection was performed using MZmine 2.53 (Pluskal et al., 2010). In the first step of the feature detection process using MZmine, each mass spectrum was processed separately to detect individual ion peaks. The *mzML* file was imported to a retention time range of 0.03–30.32 min and the mass detection noise level was 1.0E1 for MS2 with Centroid mass detector. Chromatograms were then constructed for each *m*/*z* value found in the mass list across the entire retention period. Chromatograms were built with a minimum time span of 0.01 min, a minimum height of 5.0E3, and an m/z tolerance of 0.001 m/z or 5.0 ppm. Finally, each chromatogram was deconvoluted into its individual features. Chromatogram deconvolution, which separates each detected chromatogram into individual peaks, was achieved using a baseline cut-off algorithm with a minimum peak height of 2.0E3, a peak duration range of 0.00–0.4 min, and a baseline level of 5.0E2. Deconvoluted peaks were de-isotoped using the isotopic peaks grouper algorithm with an *m*/*z* tolerance of 0.006 or 10.0 ppm and a retention time tolerance of 0.15 min. MZmine selects different algorithms for each of these steps depending on the nature of the MS data (Weng, 2020). The peaks were aligned in a peak table by joint alignment with an m/z tolerance of 0.001 or 5.0 ppm. The aligned data were statistically analyzed using MetaboAnalyst 5.0 (http://www.metaboanalyst.ca) (Pang et al., 2021).

After the matrix data were normalized, the multivariate analysis comprised principal component analysis (PCA), partial least squares discriminant analysis (PLS-DA), and the building of a heatmap showing the correlation between variables and samples. Finally, a hierarchical clustering dendrogram was obtained using a Euclidian distance measure and Ward clustering algorithm.

### Polyhydroxybutyrate (PHB) isolated from *S*. *costicola* YSL5

A 10 ml liquid culture of *S*. *costicola* YSL5 preculture was transferred to 100 ml of modified K medium in a 250 ml Erlenmeyer flask. With continuous shaking at 28°C and 180 rpm for 3 days, 10 ml of the liquid culture was inoculated in a 2 L Erlenmeyer flask containing 1.2 L of the medium (28°C, 180 rpm, 3 days, 12 L in total). The culture was extracted using ethyl acetate/water layer separation, and the ethyl acetate layer was subsequently concentrated *in vacuo*. The dried plant extracts were purified using a semi-preparative HPLC equipped with a 1,260 Infinity binary pump and YMC-triart C18 column (150 × 10.0 mm, 5 μm; YMC KOREA Co., Seongnam, Korea) with 0.1% formic acid in water (A) and 0.1% formic acid in acetonitrile (B). The conditions were 10% B (0–10 min), 10–50% B (10–30 min), 50–100% B (30–35 min), and UV detection at 210 nm. Nuclear magnetic resonance (NMR) data of the separated single compounds were recorded on a JEOL 600 MHz NMR spectrometer equipped with a 600 MHz ECZR narrow bore spectrometer. ^1^H NMR chemical shifts at *δ*_H_ 2.50 (DMSO-*d*_6_) and ^13^C NMR chemical shifts at *δ*_C_ 39.51 (DMSO-*d*_6_) were set as reference peaks, and the chemical structures were assigned by interpreting combinational data from two-dimensional (2D) NMR such as correlation spectroscopy (COSY), heteronuclear single quantum coherence (HSQC), and heteronuclear multiple bond correlation (HMBC) experiments.

### Detection in a botanical specimen

Whole parts of *S. maritima* were dried at 45°C for 1 day with a dehydrator (SHINIL, Cheonan, Korea). Ground dried samples were extracted with methanol at 35°C for 120 min using an ultrasound-assisted extraction method. Methanol was used as the extraction solvent to obtain a diverse variety of compounds from plants. The extract was concentrated *in vacuo* and dissolved in methanol to a final concentration of 250 μg/ml. Metabolite analysis was conducted with MS/Q-TOF-MS with 0.1% formic acid in water (A) and 0.1% formic acid in acetonitrile (B). The conditions were 10–100% B (0–20 min), 100% B (20.1–25 min), 100–10% B (25.1–30 min), and 10% B (30.1–35 min), with an injection volume of 10 μL and a flow rate of 0.3 ml/min. Mass spectrometry was performed using electrospray ionization (ESI) source in the positive mode. Extracted-ion chromatogram (*m*/*z* [M + H]^+^ and *m*/*z* [M + Na]^+^) and fragmentation patterns were analyzed to compare and detect compounds in both the methanol extract of *S. maritima* and liquid culture of *S*. *costicola* YSL5. The ethyl acetate extract was dissolved in methanol at a concentration of 1,000 μg/ml and the compound was isolated using a semi-preparative LC-MS system. HPLC measurements were performed using an Agilent 1,100 Series (Agilent Technologies, Santa Clara, CA, United States) equipped with a YMC-Triart C18 column (150 × 10.0 mm, 5 μm; YMC KOREA Co., Seongnam, Korea). The mobile phases were 0.1% formic acid in water (A) and 0.1% formic acid in acetonitrile (B), with the following gradients: 50–100% B (0–10 min), 100% B (10.1–15 min), 100–10% B (15.1–20 min), and 10% B (20.1–25 min). Mass spectrometry was performed using a single quadrupole mass spectrometer (Waters Micromass ZQ, Waters, Massachusetts, United States ) equipped with an ESCi multimode ionization source. The MS experiment was performed under the conditions same as the analyses of *S*. *costicola* YSL5.

## Results

### Composition analysis of metagenomics sequencing

**FIGURE 1:**
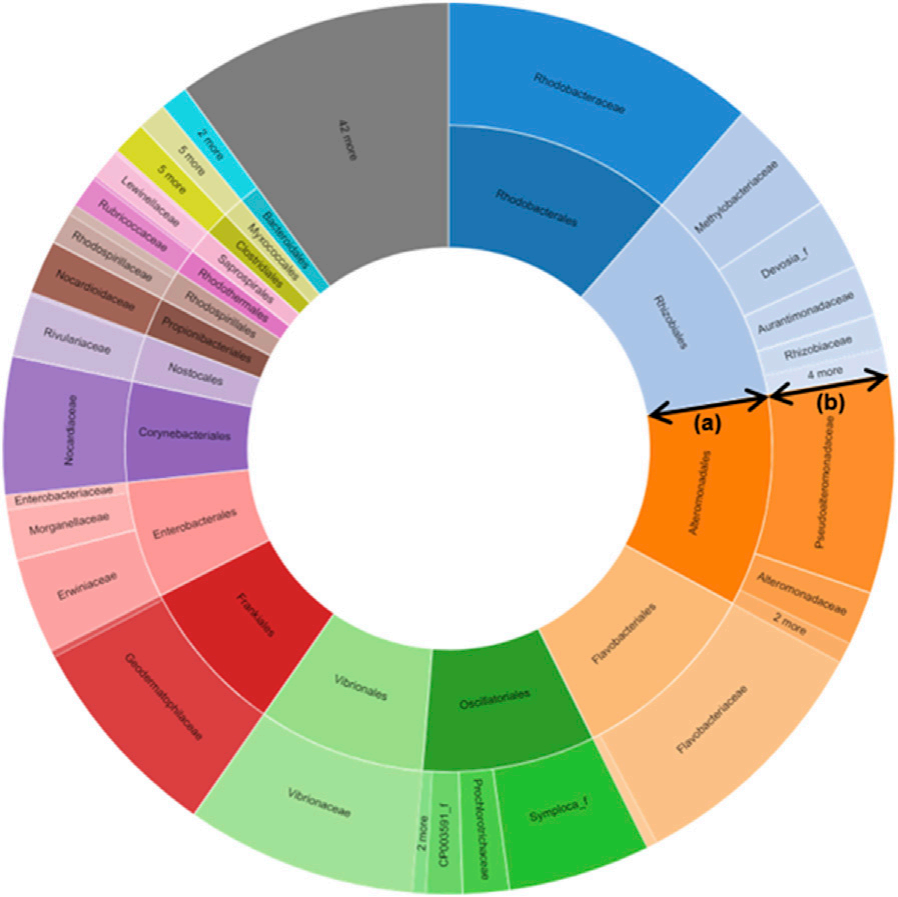
Metagenomics-targeted bacteria taxon of *Suaeda maritima* (L.) Dumort. The data shows that Vibrionaceae, the family of *Salinivibrio costicola* YSL5, was detected from *S*. *maritima.* **(A)** Order; **(B)** Family.

About 411 bp read lengths (min = 111, max = 446) of V3-V4 hypervariable regions of the bacterial 16S rRNA gene were amplified using the genomic DNA extracted from the plant material. A total of 64772 amplicons were sequenced using the Illumina MiSeq platform, and 24004 quality-filtered reads were obtained (low-quality amplicons = 1,664, non-target amplicons = 39688, chimeric amplicons = 416). The number of reads that were identified at the species level based on 97% similarity was 59942 (92.5%), and 364 bacterial species were identified. Good’s coverage, calculated as the ratio of OTUs excluding singleton in the entire read, reflects the database coverage rate of the sample, and its value for *S*. *maritima* was 99.6%, indicating that the sample reached sufficient sampling depth (cutoff = 97%, number of OTUs found in the sample = 454). The values of alpha diversity, the species diversity existing in a particular location and a particular sample, for *S*. *maritima* were 522.6 ± 29.9 for ACE, 49.0 ± 27.0 for Chao1, and 552.0 ± 0 for Jackknife. The Shannon value, an index that reflects the number of species and the effect of evenness, for *S*. *maritima* was 2.544 ± 0.028. The phylogenetic diversity of *S*. *maritima* was calculated to be 879.0 by summing the shortest distance between the system diagram’s nodes. The detected OTUs were assigned to 15 phyla (24004 OTUs in total), 38 classes, 67 orders, 127 families, 248 genera, and 383 species. Of the identified 15 phyla, the most prominent phyla were Proteobacteria (79.4%), followed by Actinobacteria (15.6%), Bacteroidetes (2.0%), and Firmicutes (1.7%). All ten major phyla have been described as the dominant taxa in soil studies. In the Proteobacteria phylum, the Gammaproteobacteria group (68.2%) was the dominant bacterial group, followed by Alphaproteobacteria and Betaproteobacteria with a total percentage of reads of 6.4 and 4.6% at the class level (Figure 1; Supplementary Tables S1–4).

### Isolation of microbes associated with *S*. *maritima* and DNA sequencing

From the fleshy leaves and roots of *S*. *maritima* on the Chitin and Czapek-Dox media, four endophytes were isolated including *Streptomyces xinghaiensis* (YSL1, GenBank accession No. OM992324), *Nocardiopsis aegyptia* (YSL2; GenBank accession No. OM992320), and two Gram-negative bacteria, *Salinivibrio costicola* (YSL5, GenBank accession No. OM992322), and *Alcaligenes aquatilis* (YSL9; GenBank accession No. ON013930).

### Analyses of secondary metabolites from *S*. *costicola* YSL5

**FIGURE 2:**
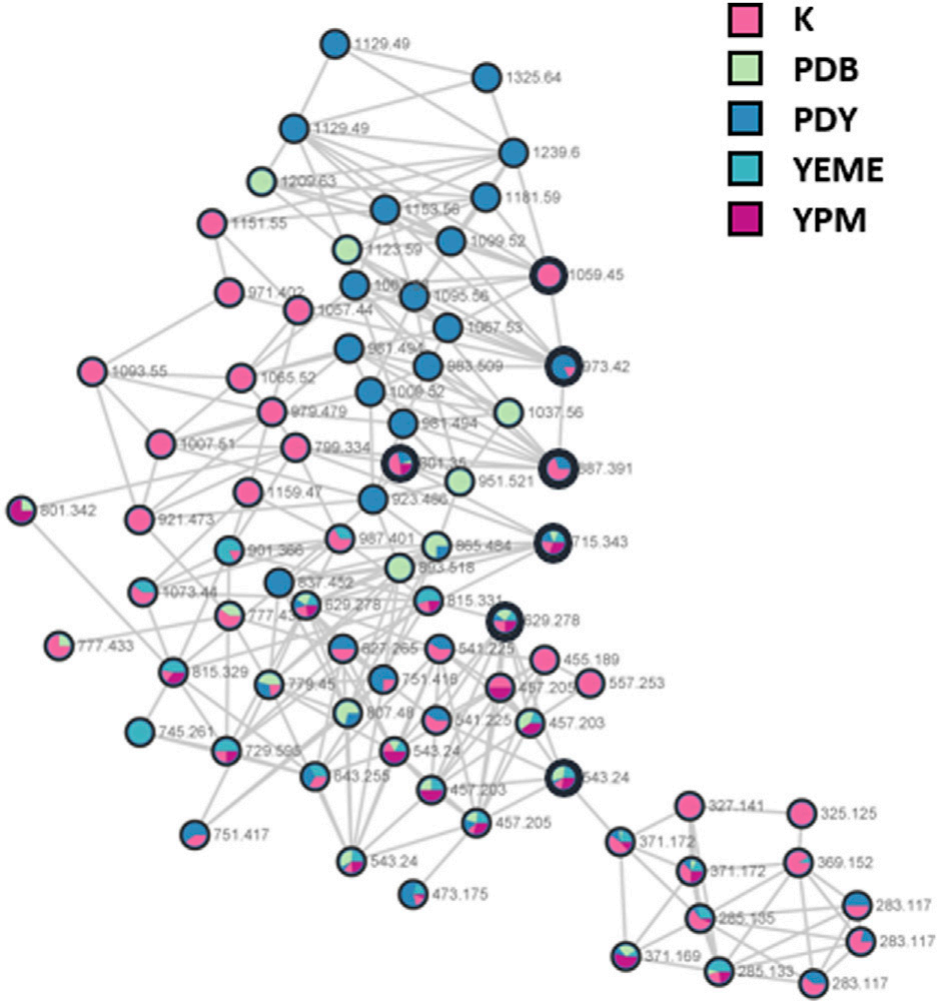
A hydroxybutyrate derivatives (bold black circles) cluster based on LC-MS/MS data of the ethyl acetate extract of *S*. *costicola* YSL5 in five broth media for 3 days. Red nodes, the metabolites produced by YSL5 in K medium; green nodes, PDB medium; blue nodes, PDY medium; light blue nodes, YEME medium; purple nodes, YPM medium.

**FIGURE 3:**
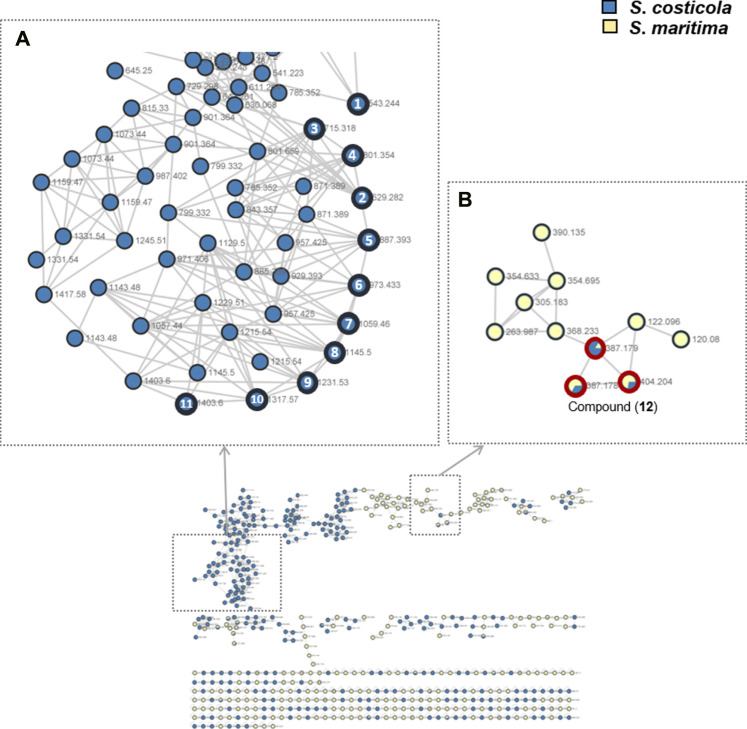
GNPS-clusters based with LC-MS/MS data of ethyl acetate extract of *S*. *costicola* YSL5 (blue nodes) and methanol extract of *S*. *maritima* (yellow nodes). **(A)** Eleven metabolites annotated as hydroxybutyrate analogs (marked with bold black circles); *m*/*z* 543.24 [M + Na]^+^ (**1**), *m*/*z* 629.28 [M + Na]^+^ (**2**), *m*/*z* 715.32 [M + Na]^+^ (**3**), *m*/*z* 801.35 [M + Na]^+^ (**4**), *m*/*z* 887.39 [M + Na]^+^(**5**), *m*/*z* 973.41 [M + Na]^+^ (**6**), *m*/*z* 1,059.46 [M + Na]^+^ (**7**), *m*/*z* 1,145.50 [M + Na]^+^ (**8**), *m*/*z* 1,231.53 [M + Na]^+^ (**9**), *m*/*z* 1,317.57 [M + Na]^+^ (**10**), and *m*/*z* 1,403.60 [M + Na]^+^ (**11**) are clustered. **(B)** One metabolite *m*/*z* 387.18 [M + H]^+^, *m*/*z* 404.20 [M + Na]^+^ (**12**) was detected both of *S*. *costicola* YSL5 extract and *S*. *maritima* extract (bold red circles).

*S*. *costicola* YSL5, an endophyte of *S*. *maritima*, was cultivated for 3 days in modified K, PDB, PDY, YEME, and YPM liquid media with sea salt (Supplementary Figure S1), and the extract was analyzed using LC-MS/MS. The diversity and quantity of secondary metabolites produced in each medium were quantified using GNPS pie charts (Figure 2). The bacterial strain *S*. *costicola* YSL5 cultured in K medium produced twelve related metabolites between *m*/*z* 543.2–1,059.4 with an increase of 86 (Supplementary Figure S3), displaying identical UV absorption (λ_max_ at 210). HRMS^2^-based analysis of the YSL5 extract clustered structurally related molecules with GNPS, and the results were visualized using Cytoscape 3.9.0. (Figure 3). The clustered nodes showed eleven analogs and other metabolites (Figure 3A). Eleven analogs: *m*/*z* 543.2 [M + Na]^+^ (1), *m*/*z* 629.2 [M + Na]^+^ (2), *m*/*z* 715.3 [M + Na]^+^ (3), *m*/*z* 801.3 [M + Na]^+^ (4), *m*/*z* 887.3 [M + Na]^+^ (5), *m*/*z* 973.4 [M + Na]^+^ (6), *m*/*z* 1,059.4 [M + Na]^+^ (7), *m*/*z* 1,145.5 [M + Na]^+^ (8), *m*/*z* 1,231.5 [M + Na]^+^ (10), *m*/*z* 1,317.5 [M + Na]^+^ (11), *m*/*z* 1,403.6 [M + Na]^+^ (12) made a large cluster.

**FIGURE 4:**
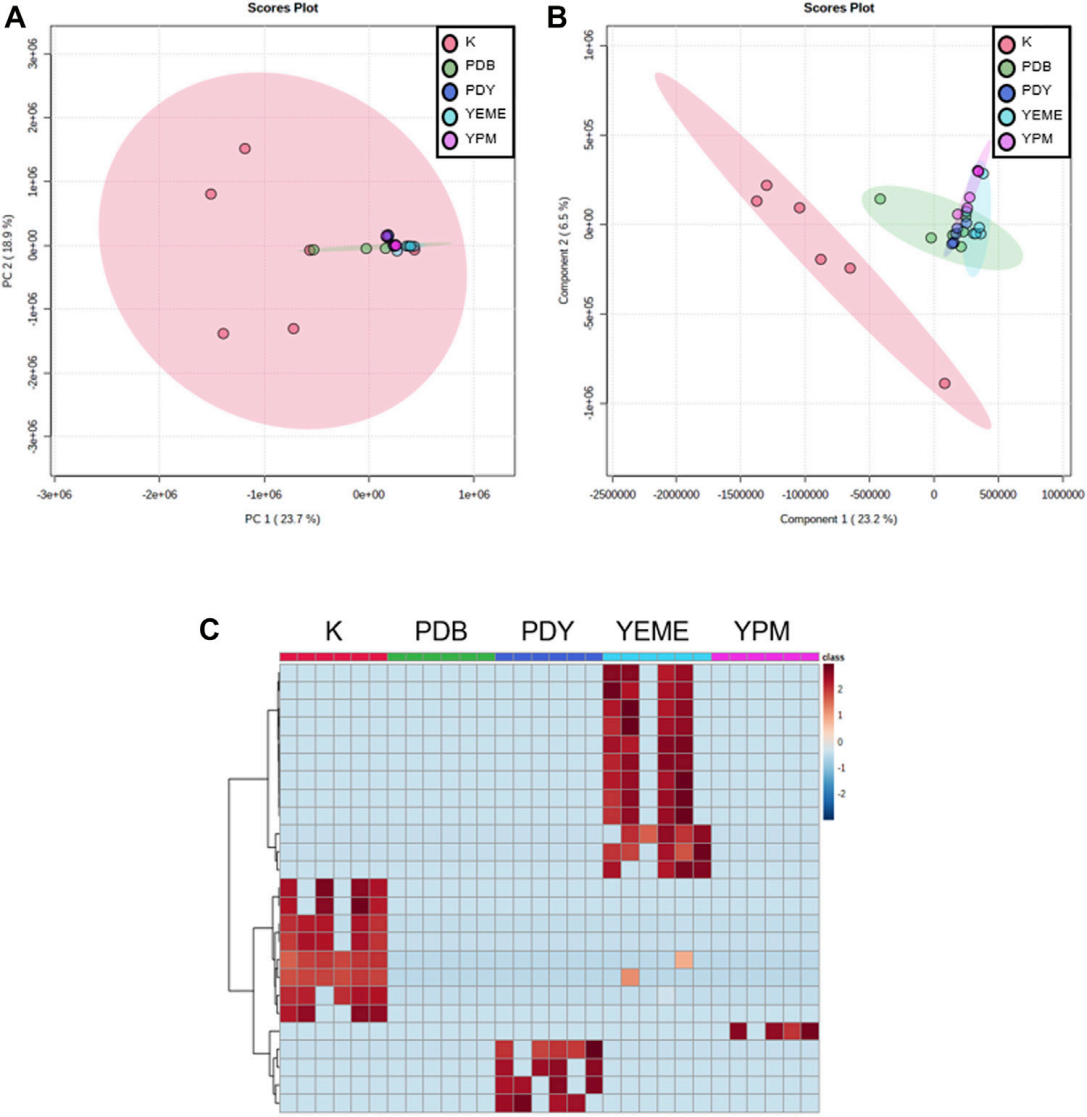
Metabolic profiling obtained using LC-MS of secondary metabolites produced by *S*. *costicola* YSL5 in five broth media with sea salt. **(A)** Partial least square discriminant analysis (PCA) plot based on the metabolic statistics analysis with the data obtained using LC-MS. **(B)** Partial least square discriminant analysis (PLS-DA) score plot based on the metabolic statistics analysis. PLS-DA model shows metabolic profile differences among metabolites produced by *S*. *costicola* YSL5 in K broth medium and metabolites produced in other four broth media **(C)** Heat map of the top 25 differential metabolites selected based on analysis of variance. Red colors indicate upregulation; blue colors indicate downregulation.

**FIGURE 5:**
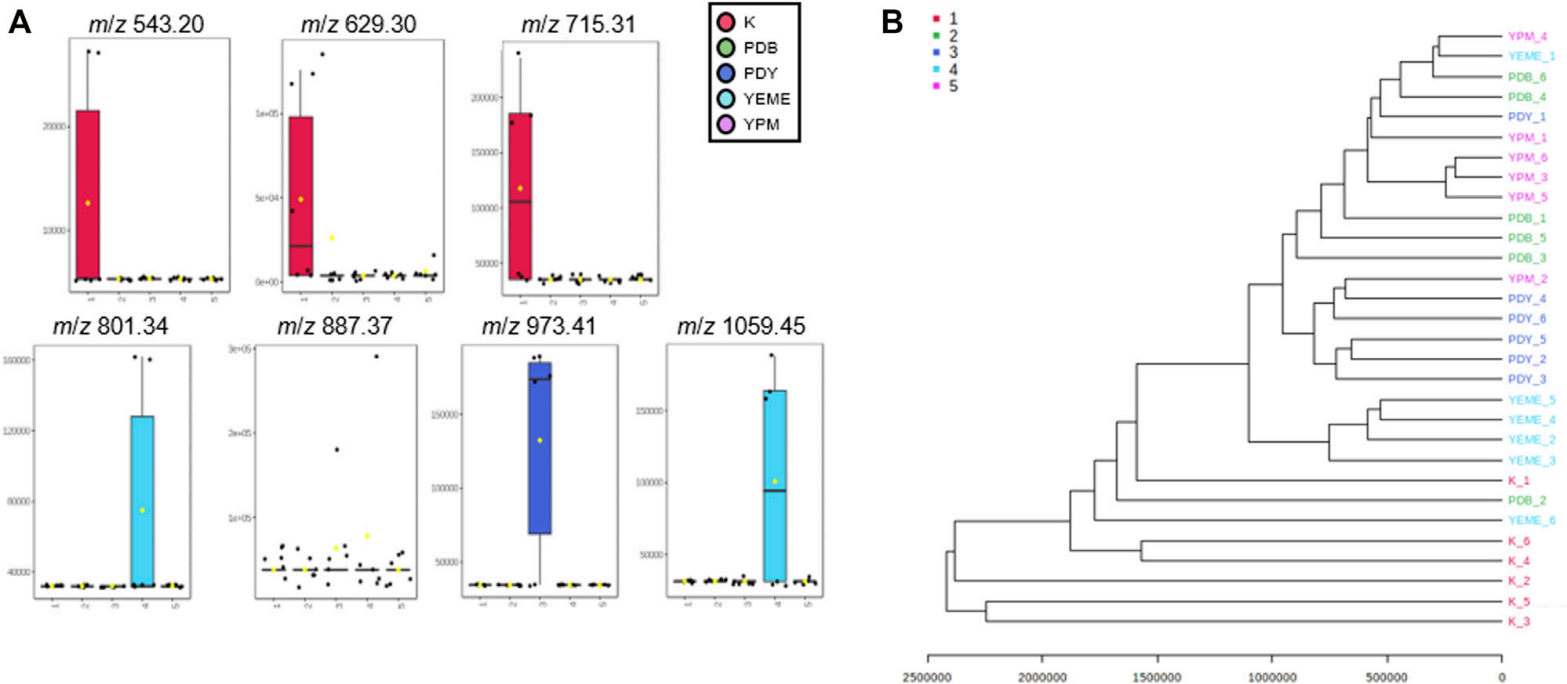
**(A)** Box and whisker plots of the normalized values of seven metabolites produced by *S*. *costicola* YSL5 in K broth medium with sea salt. Seven metabolites were *m*/*z* 543.20 [M + Na]^+^, *m*/*z* 629.30 [M + Na]^+^, *m*/*z* 715.31 [M + Na]^+^, *m*/*z* 801.34 [M + Na]^+^, *m*/*z* 887.37 [M + Na]^+^, *m*/*z* 973.41 [M + Na]^+^, and *m*/*z* 1,059.45 [M + Na]^+^. The metabolites increase *m*/*z* by 86 **(B)** Hierarchical clustering dendrogram based on the metabolic statistics analysis. The distance measure was Euclidean and the dendrogram was clustered with the Ward algorithm. The dendrogram shows distances among metabolites produced by *S*. *costicola* in five broth media.

Preprocessing was performed for statistical processing with data from six replicates. After necessary data preprocessing, a total of 971 metabolites in positive ion mode were identified from the raw data using the LC-MS/MS system. The covariance for the first five components was evaluated using unsupervised PCA. The explained variance of compounds produced by YSL5 in each of the five different media (K, PDB, PDY, YEME, and YPM) was 23.7% (PC1) and 18.9% (PC2), with a total variance of 42.6% (Figure 4A). The discrimination among the five groups was better represented by PLS-DA (covariance of 29.7%; Figure 4B), and the PLS-DA model classified K medium and others. Box and whisker plots (Figure 5A) represent differences in the relative abundance of metabolites, with *m*/*z* increasing by 86 among the media. A heatmap was generated based on the top 25 differentially expressed metabolites with the highest statistical difference and also exhibited distinct patterns of metabolites produced by *S*. *costicola* YSL5 in the five bacterial culture media (Figure 4C) (Pryke et al., 2007). Box and whisker plots of the normalized values of seven metabolites produced by *S*. *costicola* YSL5 in K broth with sea salt (Figure 5A) (Hssaini et al., 2020). The hierarchical clustering dendrogram shows similarities between the secondary metabolites produced in each medium (Figure 5B). With the PCA, PLS-DA, heatmap, and hierarchical clustering dendrogram, it can be observed that there are differences between the secondary metabolites produced in each medium. The K medium had particularly different metabolites, and the other four media showed more similarities among them than the K medium.

### Polyhydroxybutyrate (PHB) derivatives from *S*. *costicola* YSL5

2-hydroxy-3.7,11,15-tetramethyl-5.9,13,17-tetraoxo-4.8,12,16-tetraoxaicosan-19-yl 3-hydroxybutanoate **(1)** was obtained at *t*_R_ 33 min using semi-preparative HPLC. Compound **1** was isolated as a yellow oil; UV (MeOH) λ_max_ (log ε) 218 and 290 nm. The molecular formula of compound **1** was determined to have a molecular formula of C_24_H_40_O_12_Na, based on high-resolution electrospray ionization (HR-ESI) mass spectrometry, which indicated five degrees of unsaturation with a molecular ion at *m*/*z* 543.2481 [M + Na]^+^. The structure was confirmed by ^1^H, ^13^C NMR, and 2D data (Supplementary Figure S4–8).

2-hydroxy-3.7,11,15-tetramethyl-5.9,13,17-tetraoxo-4.8,12,16-tetraoxaicosan-19-yl 3-hydroxybutanoate (**1**): ^1^H NMR (600 MHz, DMSO-*d*_*6*_) δ_H_ 5.17-5.05 (5H, m), 4.75 (1H, br s), 4.67 (2H, qt, *J* = 6.5), 3.95 (1H, q, *J* = 6.5), 3.60 (1H, m), 2.64-2.51 (9H, m), 2.32 (1H, dd, *J* = 14.5), 2.26 (1H, dd, *J* = 14.5), 1.20 (2H, s), 1.19 (3H, d, *J* = 1.4), 1.19-1.18 (9H, m), 1.17 (3H, d, *J* = 1.2)

^13^C NMR (150 MHz, DMSO-*d*_*6*_) five carbonyl carbons [δ_C_ 170.25, 169.36, 169.05, 168.99, 168.98], four methoxy and two alcohol carbons [δ_C_ 74.82, 73.77, 67.40, 67.23, 67.18, 66.69, 63.28], five methylene carbons [δ_C_ 44.06, 40.21, 40.10, 40.01, 39.99], seven methyl carbons [δ_C_ 23.21, 19.39, 19.37, 19.31 (2C), 18.34, 15.05]; HR-ESI-MS m/z 543.2481 [M + Na]^+^ (calcd. for C_24_H_40_O_12_Na, 543.2423).

PHB, a similar structure to 2-hydroxy-3.7,11,15-tetramethyl-5.9,13,17-tetraoxo-4.8,12,16-tetraoxaicosan-19-yl 3-hydroxybutanoate (**1**), is known to be an energy source for bacteria and plays important role in plant growth (Okon and Itzigsohn, 1992; Reddy et al., 2019). However, the mechanism of PHB as a plant growth-promoting factor remains unclear (Trainer and Charles, 2006). The endophytic bacterium *Herbaspirillum seropedicae* produces PHB, which is stored in insoluble granules. PHB production showed that *H*. *seropedicae* significantly increased the growth of a variety of plants, including rice, corn, and sugarcane. In that study, *Setaria viridis* and five mutants in the PHB metabolism of *H*. *seropedicae* revealed several results regarding the importance of PHB for bacterial colonization in *S*. *viridis* roots (Alves et al., 2019). In another study, PHB-producing *Pseudomonas fluorescens* was isolated from the leaves of abiotic stress-tolerant Pangola grass. *P*. *fluorescens* is known as a plant growth-promoting bacterium, and a study revealed that a novel extracellular enzyme related to PHB production could have positive effects on plant growth (Stritzler et al., 2018).

### Detection in a botanical specimen

**FIGURE 6:**
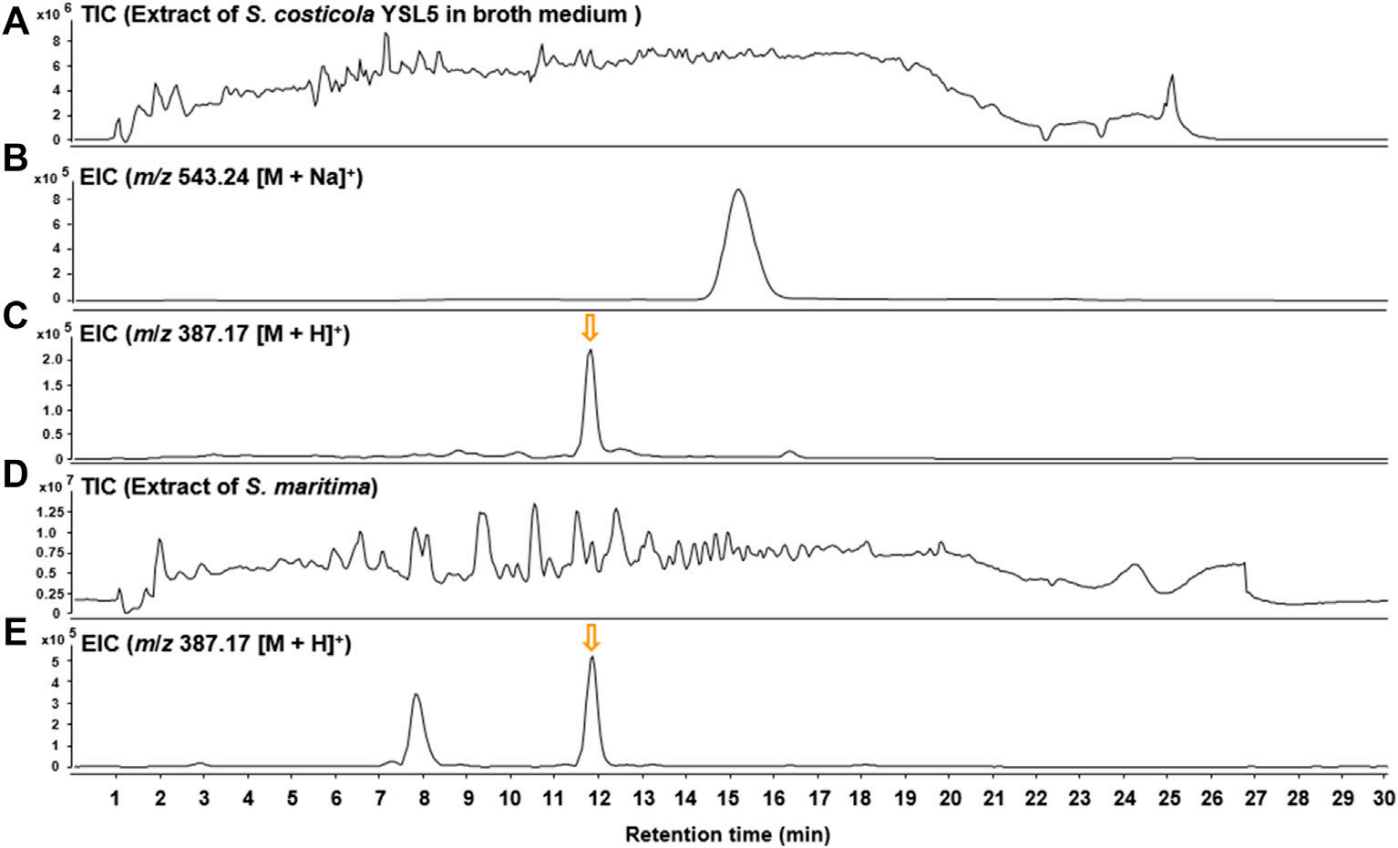
LC-MS chromatograms of m/*z* 387.17 [M + H]^+^ were obtained from the extract of *S. costicola* YSL5 in broth medium and *S*. *maritima* in positive ion mode. **(A)** Total ion current (TIC) chromatogram of the ethyl acetate extract of the *S. costicola* YSL5 liquid culture broth. **(B)** Extracted-ion chromatogram (EIC) of the *S. costicola* YSL5 liquid broth (2-hydroxy-3.7,11,15-tetramethyl-5.9,13,17-tetraoxo-4.8,12,16-tetraoxaicosan-19-yl 3-hydroxybutanoate, *m*/*z* 387.17 [M + H]^+^). **(C)** Extracted-ion chromatogram (EIC) of the *S. costicola* YSL5 liquid broth (polyhydroxybutyrate derivatives, *m*/*z* 387.17 [M + H]^+^). **(D)** Total ion current (TIC) chromatogram of the methanol extract of *S. maritima*. **(E)** Extracted-ion chromatogram (EIC) of *S. maritima* (polyhydroxybutyrate derivatives, *m*/*z* 387.17 [M + H]^+^). The arrows indicate the exact elution times of the extracted ions.

Based on the metagenomic analysis, *S*. *costicola* YSL5 is thought to be an endophyte of *S*. *maritima*. To provide additional evidence, we investigated whether the metabolites produced by *S*. *costicola* YSL5 accumulated in *S*. *maritima*. *S*. *maritima* methanol extract was analyzed by LC-MS/MS under the same conditions as *S*. *costicola* YSL5 extract. The cluster showed that PHB derivatives (**12**) (*m*/*z* 387.1 [M + H]^+^, *m*/*z* 404.2 [M + Na]^+^) were detected in both *S*. *costicola* YSL5 culture broth extract and *S*. *maritima* methanol extract using GNPS analysis (Figure 3B). In the LC-MS chromatogram, EIC (*m*/*z* 387.1 [M + H]^+^) demonstrated that a compound (12) was detected at the same retention time and mass pattern as YSL5 (Figures 6C, E). Compound 12 was isolated as an oil with a molecular ion at *m*/*z* 387.1787 [M + H]^+^ at t_R_ = 6 min by semi-preparative LC-MS. Based on ^1^H, COSY, HSQC, HMBC, and NOESY data, the molecular formula of compound 12 was determined to be a PHB derivative (Supplementary Figure S9–11).

## Discussion

Plant endophytes utilize the endosphere of plants to stimulate plant development, elicit a defense response against pathogen attack, and mitigate abiotic stresses induced by drastic changes in extreme climates under harsh conditions. Although salinity is one of the most influential environmental factors on microbial evolution and community composition, little is known about the ecological functioning of microbial communities associated with halophytes growing in extremely saline environments such as seawater, salterns, salt mines, and mud flats. They have developed distinct morphological, anatomical, and physiological mechanisms to thrive in high-salt conditions during the course of evolution (Grigore et al., 2014). Microorganisms living in a high-salinity environment must consider the high energy loss in metabolic processes due to insufficient energy sources (Oren, 2008). The metabolic mechanisms that reduce the energy required to sustain life in microorganisms are encouraged. These mechanisms are characterized by modest growth rates, and the majority of the organism’s energy is directed towards maintenance-related activities (Shoemaker et al., 2021). These activities include osmotic equilibration, oxygen stress defense, motility, and more sustainable metabolic pathways. For instance, certain types of bacteria can store lipid droplets within their cells in the form of polyhydroxyalkanoates, triglycerides, or wax esters when subjected to stress (Alvarez et al., 1997; Wältermann and Steinbüchel, 2005; Thomas et al., 2019).

In this study, we used metagenomics analysis to track the bacterial endophytes of *S*. *maritima* and their secondary metabolites to advance plant microbiome research. Also, we used it to identify specific bacterial strains that generate biologically active or functional secondary metabolites that accumulate in host plants. When constructing agricultural systems for progressive and sustainable agricultural production, information regarding the association between plants and the microbial population in the soil would be useful, and this knowledge may be gained through the application of metagenomics. The utilization of metagenomic techniques to investigate soils that have been infected with organic manure would be beneficial when designing strategies for fertilization and would lessen the reliance of farmers on chemical fertilizers (Manjula and Narsimha, 2015). Goel et al. reported that metagenomics has the potential to be utilized for the development of sustainable agriculture. These programs can constantly evaluate microorganisms that are beneficial for plant growth (Goel et al., 2017). Similarly, functional metagenomics can be used to reshape and redirect rhizosphere microbial activity (Louca et al., 2018). Investigations into metagenomics, when combined with assessments of efficiency, may thus assist in improving bioponic systems and urban agriculture, such as smart farms, in a way that is more environmentally friendly (Bao et al., 2021). To achieve sustainable agriculture on a global scale and to reclaim soil through phytoremediation and bioremediation, a growing number of countries are now utilizing rhizospheric bacteria that are capable of producing plant growth promoters in the presence of salt stress (Tank and Saraf, 2010). Our investigation of the bacteria isolated from *S*. *maritima* resulted in the identification of four bacterial strains (YSL1, 2, 5, and 9), with YSL5 being the only strain matching the metagenomic results of *S*. *maritima* at the family level. While moderate salinity of 15% is generally regarded to be a transitional salinity for marine microbes and moderate halophiles, the bacterial strain YSL5 grew in a non-saline bacterial culture medium. We cultivated YSL5 in diverse bacterial cultivation media to investigate secondary metabolites by upregulating the growth of the bacterium, and identified major secondary metabolites, PHB, using spectroscopic analysis. The crude extracts of YSL5 were compared with methanol extracts of *S*. *maritima* through GNPS analysis to detect accumulated secondary metabolites in the host produced by the endophytic bacterium YSL5. Using LC-MS and GNPS chemical profiling, two secondary metabolites were detected at identical retention times, showing the identical MS/MS patterns (*m*/*z* 387.1787 [M + H]^+^, *m*/*z* 404.2029 [M + Na]^+^). After cultivating YSL5 on a large scale, the metabolite was identified as PHB with molecular weight (*m*/*z* 387.1787 [M + H]^+^). PHB, a high-molecular-weight polyester, is found in plants as a plant growth-promoting agent and accumulates as storage carbon in many bacterial species. In a hyperosmotic environment, PHB in the cytoplasm promotes the survival of bacterial cells exposed to osmotic stress. Although the mechanism of its protective action is unknown, the primary strategy of most bacteria under hyperosmotic stress is the accumulation of a diverse range of compatible solutes. The ability to adapt to changes in external osmolarity is critical for survival. Endophytic bacteria have developed various strategies to deal with osmotic pressure and changes in extracellular osmolarity in halophytes. Owing to the high osmotic potential of the soil solution and nutrient absorption limitations resulting from an oversupply of sodium and chloride ions, the high salinity of the water directly and effectively inhibits plant development (Sairam and Tyagi, 2004). Nutrient uptake inadequacies are induced by an excess of sodium and chloride in the soil solution (Nabti, 2015). Halophytes and halotolerant rhizospheric and endophytic microorganisms are uniquely suited to combat saline stress conditions. Functional interactions between halotolerant hosts and endophytic microorganisms can reduce the effects of salt pressure in the rhizosphere (Biró et al., 2002). In addition, the halophilic or halotolerant plant growth-promoting bacteria are characterized by their capacity to stimulate anti-oxidant enzymes that are involved in the tolerance of plants against salt stress (Kohler et al., 2009). According to Kadouri et al., compounds including PHB may also play a role in the resistance of *Azospirillum brasilense* to osmotic stress and root colonization (Kadouri et al., 2003). Arora et al. reported that the maximum PHB content was detected in salt-tolerant strains at higher salt concentrations, which defines the critical function of PHB in cell protection in saline environments, while the halotolerant strains accumulated the least amount of PHB when the salinity was either low or nil. However, the role of the accumulated PHB remains unclear (Arora et al., 2006). Based on the hypothesis regarding PHB accumulation in the host plant, we observed that *S. costicola* YSL5 produces PHB derivatives in the host halophyte *S. maritima*. These PHB derivatives potentially interact with one another to counter the osmotic stress, suggesting that PHB plays a role in cell protection under saline conditions.

In conclusion, we explored the bacterial communities associated with the halophyte plant *S*. *maritima* using metagenomics and functionally associated traits of the endophytic bacterium YSL5 and its secondary metabolites. The results from this study may form the basis for the future development of new natural product discovery and their function using metagenomic techniques. Furthermore, this study demonstrates that halophytes in high-salinity environments, such as salterns, are an enormous and untapped resource of new microorganisms that could have practical benefits in environments with a high level of salinity.

## Data Availability

The datasets presented in this study can be found in online repositories. The names of the repository/repositories and accession number(s) can be found below: https://www.ncbi.nlm.nih.gov/, OM992322; https://www.ncbi.nlm.nih.gov/, OM992320; https://www.ncbi.nlm.nih.gov/, OM992324; https://www.ncbi.nlm.nih.gov/, ON013930.
