# Supplementary material for: Metabolomic analysis of halotolerant endophytic bacterium Salinivibrio costicola isolated from Suaeda maritima (L.) dumort
Source: Front Mol Biosci. 2022 Sep 2;9:967945. doi: 10.3389/fmolb.2022.967945 (PMC9478568; doi:10.3389/fmolb.2022.967945)
Supplement: Supplementary file 1 [file DataSheet1.pdf]

Supplementary Material

Metabolomic of Halotolerant Endophytic Bacterium *Salinivibrio costicola* Isolated from *Suaeda maritima* (L.) Dumort.

Jaeyoun Lee [1†], Soohyun Um[1†], Seung Hyun Kim[1,*]

[1] College of Pharmacy, Yonsei Institute of Pharmaceutical Sciences, Yonsei University, Incheon 21983, South Korea

Corresponding authors:
*(S.H.K.) Tel: +82-32-749-4514. E-mail: kimsh11@yonsei.ac.kr

Table of Contents

S3 : **Supplementary Figure 1.** *Salinivibrio costicola* YSL5 cultured on modified K solid medium.
Supplementary Figure 2. A comparison of five different culture media.

S4 : **Supplementary Figure 3.** Total ion current (TIC) chromatogram and Extracted-ion chromatogram (EIC) of the metabolites detected from the liquid broth of *S. costicola* YSL5

S5 : **Supplementary Figure 4.** ^{1}H NMR spectrum (600 MHz) of 2-hydroxy-3,7,11,15-tetramethyl-5,9,13,17-tetraoxo-4,8,12,16-tetraoxaicosan-19-yl 3-hydroxybutanoate in DMSO-d_6.

S6 : **Supplementary Figure 5.** COSY NMR spectrum (600 MHz) of 2-hydroxy-3,7,11,15-tetramethyl-5,9,13,17-tetraoxo-4,8,12,16-tetraoxaicosan-19-yl 3-hydroxybutanoate in DMSO-d_6.
Supplementary Figure 6. HMBC NMR spectrum (600 MHz) of 2-hydroxy-3,7,11,15-tetramethyl-5,9,13,17-tetraoxo-4,8,12,16-tetraoxaicosan-19-yl 3-hydroxybutanoate in DMSO-d_6.

S7 : **Supplementary Figure 7.** HSQC NMR spectrum (600 MHz) of 2-hydroxy-3,7,11,15-tetramethyl-5,9,13,17-tetraoxo-4,8,12,16-tetraoxaicosan-19-yl 3-hydroxybutanoate in DMSO-d_6.
Supplementary Figure 8. ROESY NMR spectrum (600 MHz) 2-hydroxy-3,7,11,15-tetramethyl-5,9,13,17-tetraoxo-4,8,12,16-tetraoxaicosan-19-yl 3-hydroxybutanoate in DMSO-d_6.

S8 : **Supplementary Figure 9.** ^{1}H NMR spectrum (600 MHz) of polyhydroxybutyric acid derivatives in DMSO-d_6.

S9 : **Supplementary Figure 10.** COSY NMR spectrum (600 MHz) of polyhydroxybutyric acid derivatives in DMSO-d_6.
Supplementary Figure 11. HMBC NMR spectrum (600 MHz) of polyhydroxybutyric acid derivatives in DMSO-d_6.

S10 : **Supplementary Table 1.** Taxon Composition List of *S. maritima* - Phylum
Supplementary Table 2. Taxon Composition List of *S. maritima* - Class

S11 : **Supplementary Table 3.** Taxon Composition List of *S. maritima* - Order

S12-13 : **Supplementary Table 4.** Taxon Composition List of *S. maritima* - Family

1.1 Supplementary Figures

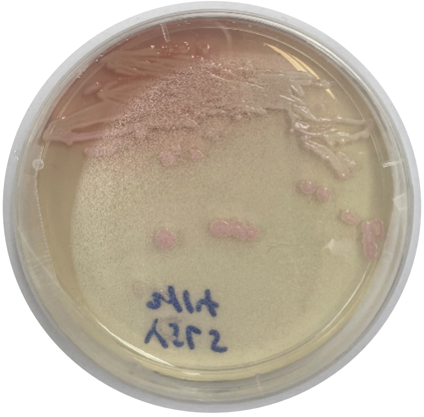

Supplementary Figure 1. *Salinivibrio costicola* YSL5 cultured on modified K solid medium with sea salt for 4 days.

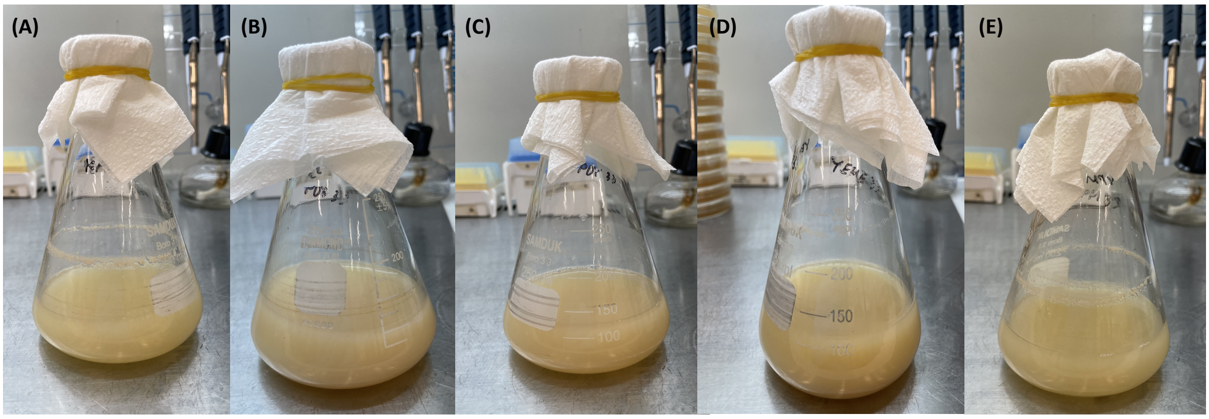

Supplementary Figure 2. A comparison of five different culture media. (A) A culture in K broth with sea salt of *S. costicola* YSL5 for 3 days. (B) A culture in PDB broth with sea salt for 3 days. (C) A culture in PDY broth with sea salt for 3 days (D) A culture in YEME broth with sea salt for 3 days. (E) A culture in YPM broth with sea salt for 3 days.

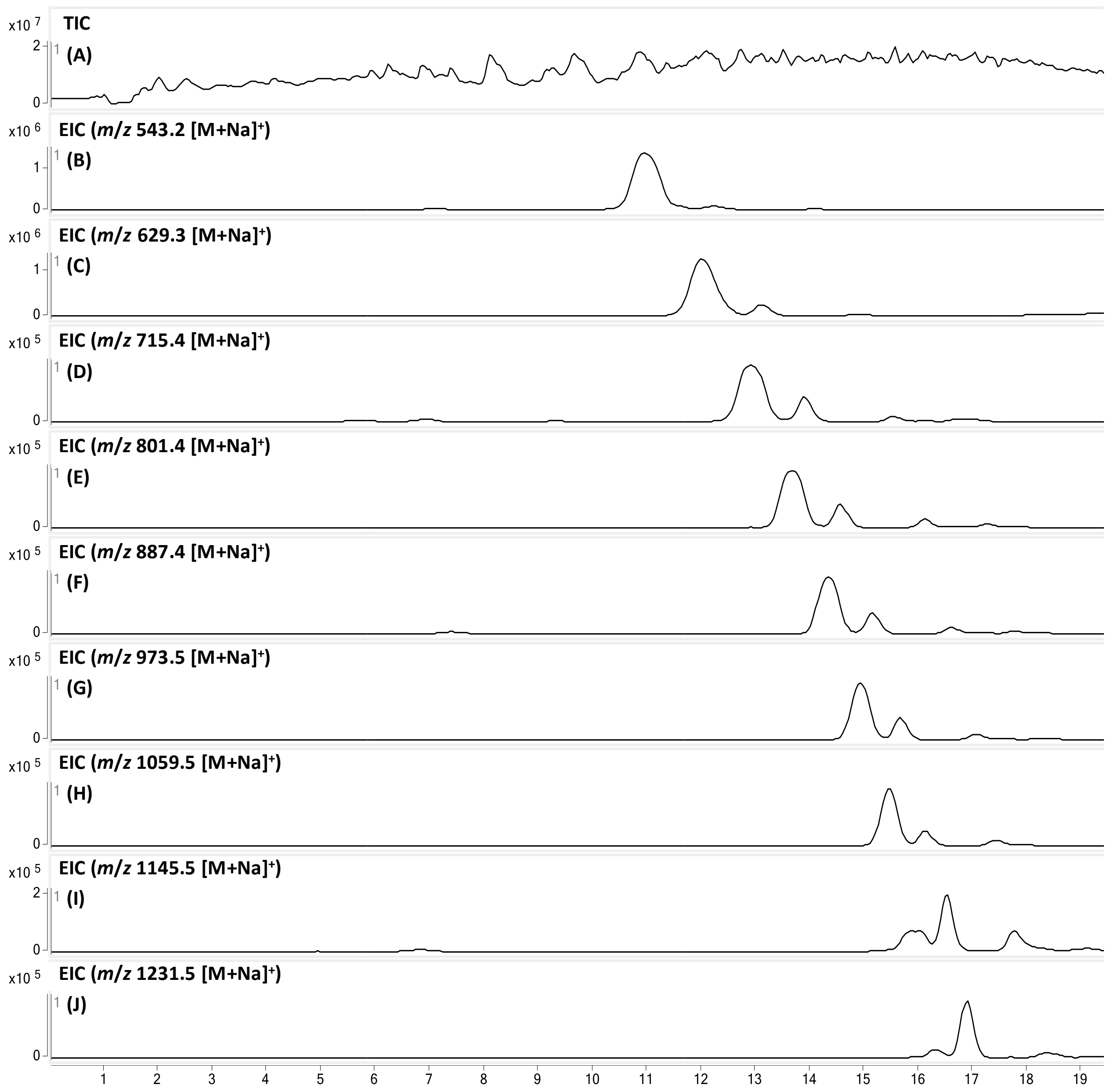

Supplementary Figure 3. (A) Total ion current (TIC) chromatogram of liquid culture broth of *S. costicola* YSL5. (B) Extracted-ion chromatogram (EIC) of *m/z* 543.2 $[M+Na]^+$, (C) EIC of *m/z* 629.3 $[M+Na]^+$, (D) EIC of *m/z* 715.3 $[M+Na]^+$, (E) EIC of *m/z* 801.4 $[M+Na]^+$, (F) EIC of *m/z* 887.4 $[M+Na]^+$, (G) EIC of *m/z* 973.5 $[M+Na]^+$, (H) EIC of *m/z* 1059.5 $[M+Na]^+$, (I) EIC of *m/z* 1145.5 $[M+Na]^+$, (J) EIC of *m/z* 1231.5 $[M+Na]^+$

Supplementary Figure 4. ^{1}H NMR spectrum (600 MHz) of 2-hydroxy-3,7,11,15-tetramethyl-5,9,13,17-tetraoxo-4,8,12,16-tetraoxaicosan-19-yl 3-hydroxybutanoate in DMSO-d_6.

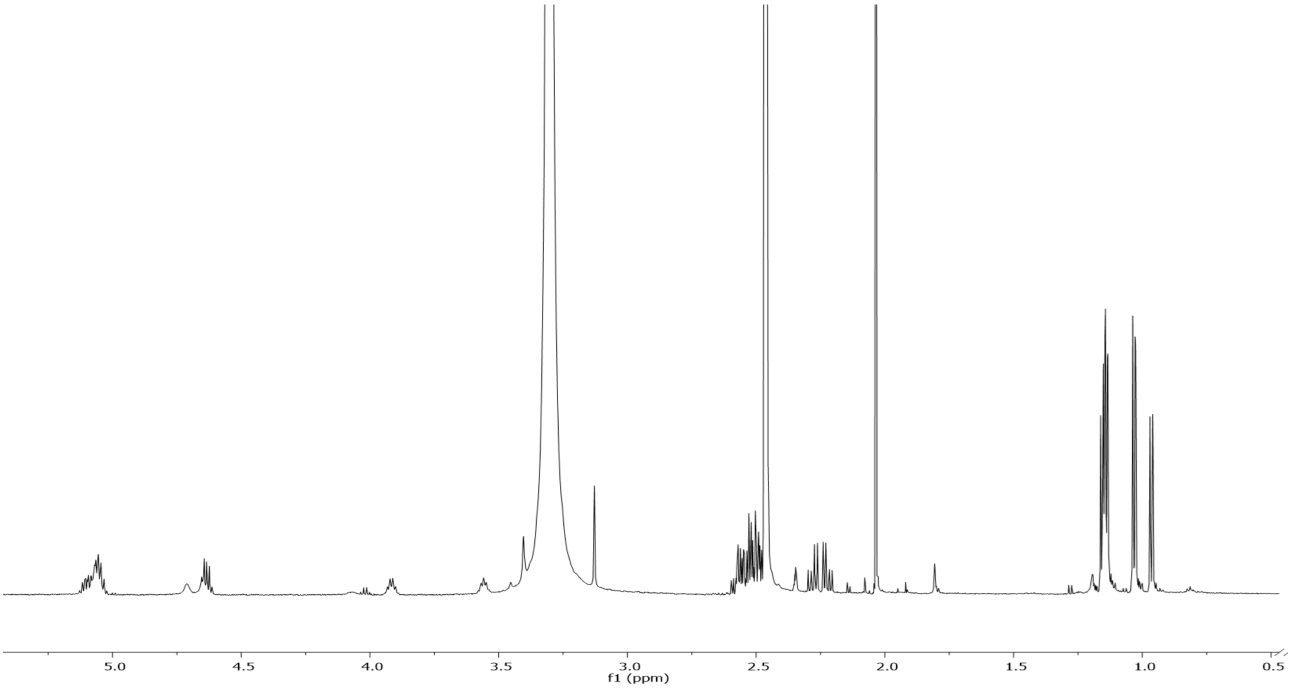

Supplementary Figure 5. COSY NMR spectrum (600 MHz) of 2-hydroxy-3,7,11,15-tetramethyl-5,9,13,17-tetraoxo-4,8,12,16-tetraoxaicosan-19-yl 3-hydroxybutanoate in in DMSO-d_6.

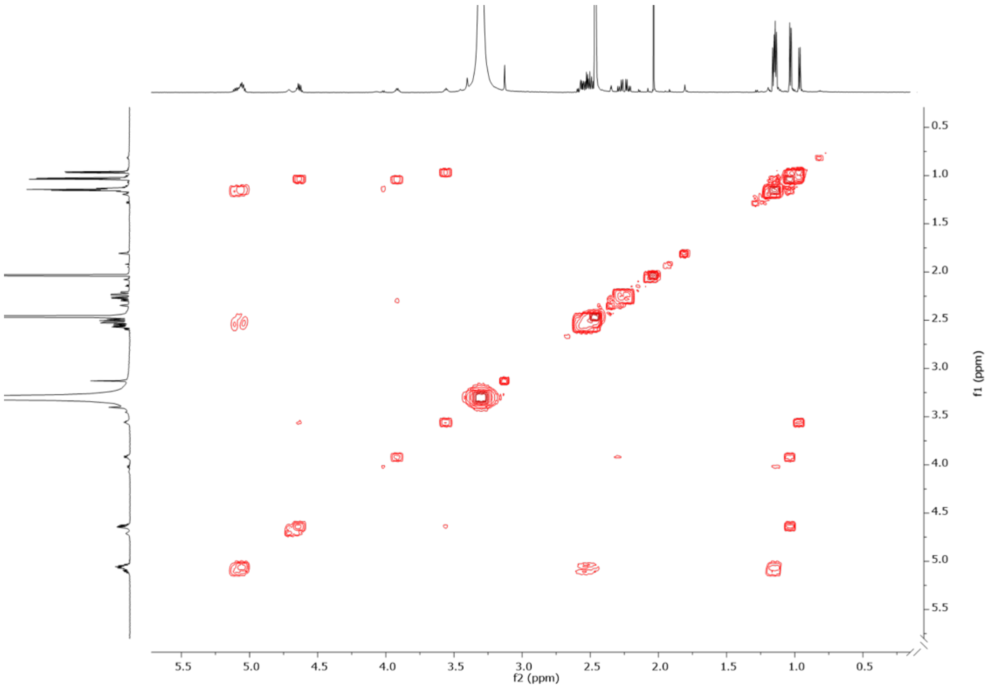

Supplementary Figure 6. HSQC NMR spectrum (600 MHz) of 2-hydroxy-3,7,11,15-tetramethyl-5,9,13,17-tetraoxo-4,8,12,16-tetraoxaicosan-19-yl 3-hydroxybutanoate in in DMSO-d_6.

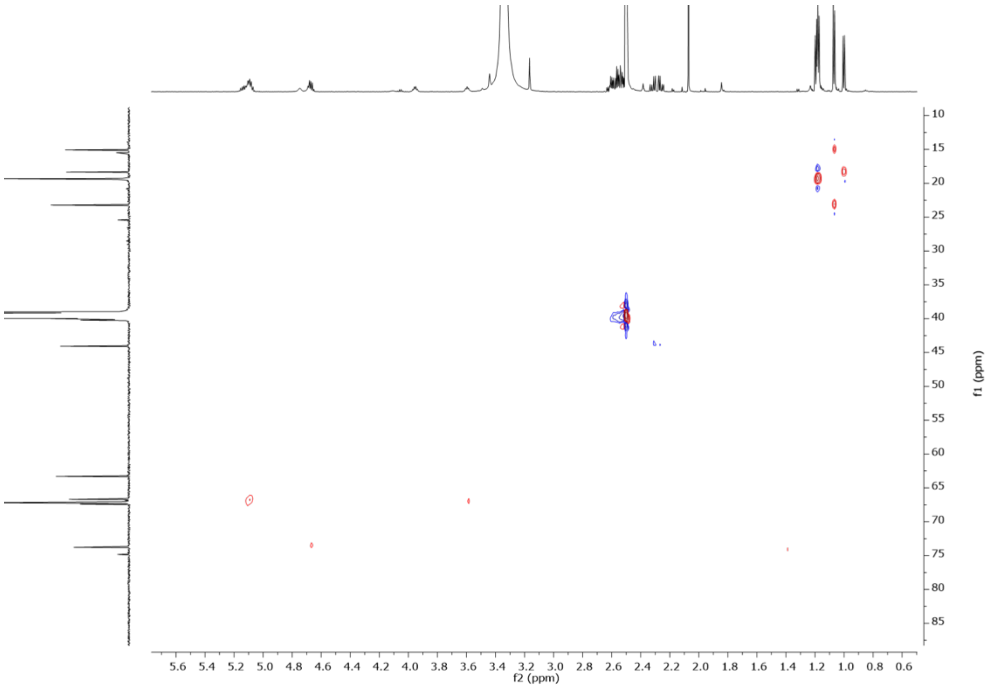

Supplementary Figure 7. HMBC NMR spectrum (600 MHz) of 2-hydroxy-3,7,11,15-tetramethyl-5,9,13,17-tetraoxo-4,8,12,16-tetraoxaicosan-19-yl 3-hydroxybutanoate in in DMSO-d_6.

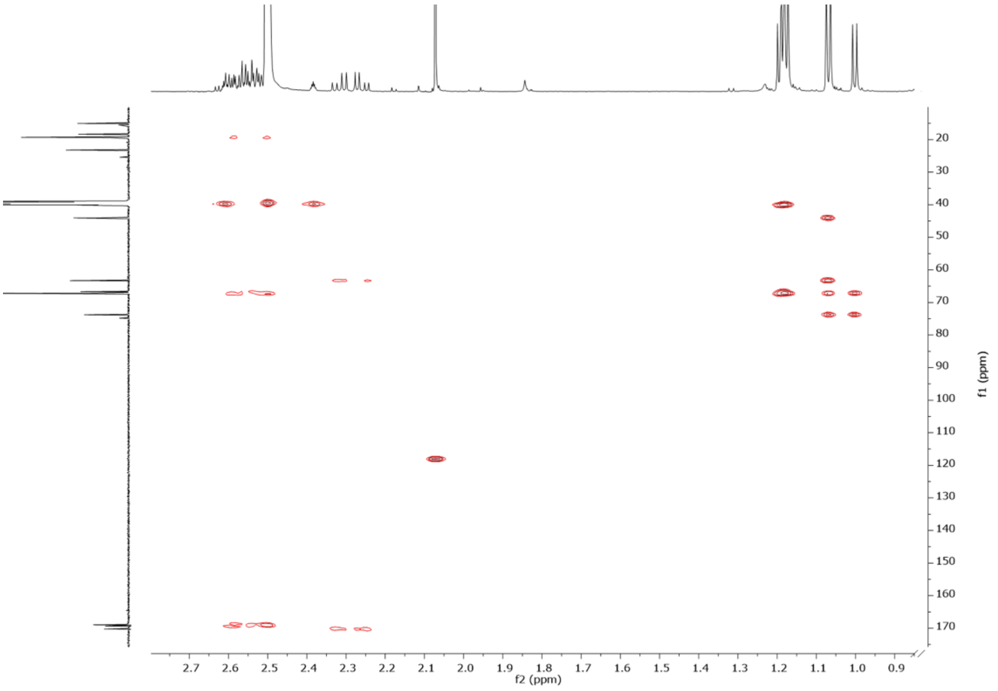

Supplementary Figure 8. ROESY NMR spectrum (600 MHz) of 2-hydroxy-3,7,11,15-tetramethyl-5,9,13,17-tetraoxo-4,8,12,16-tetraoxaicosan-19-yl 3-hydroxybutanoate in in DMSO-d_6.

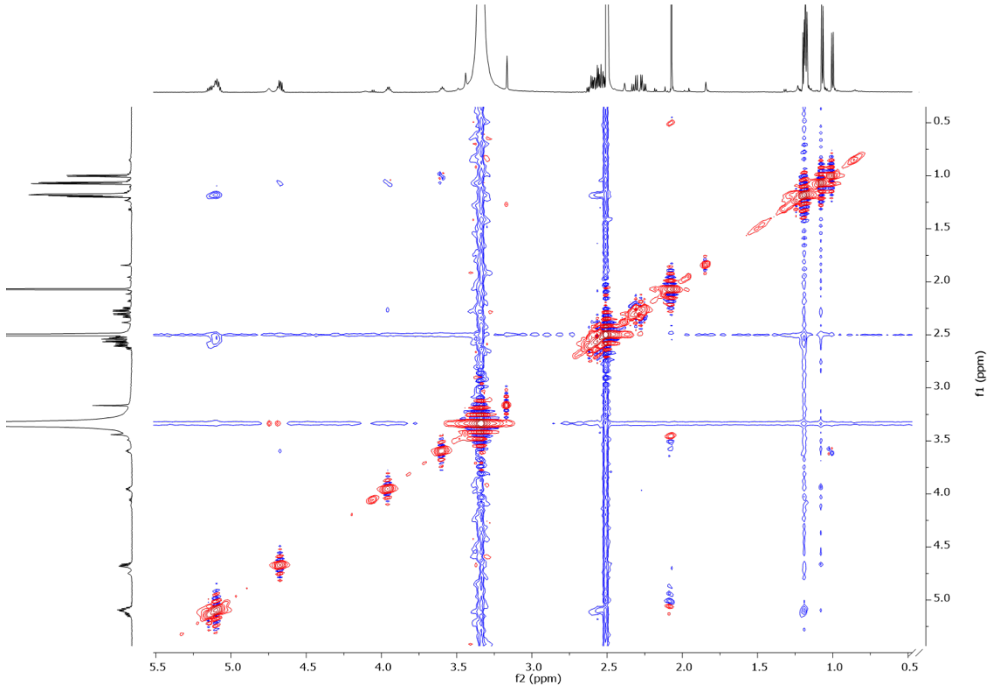

Supplementary Figure 9. ^{1}H NMR spectrum (600 MHz) of polyhydroxybutyric acid derivatives in DMSO-d_6.

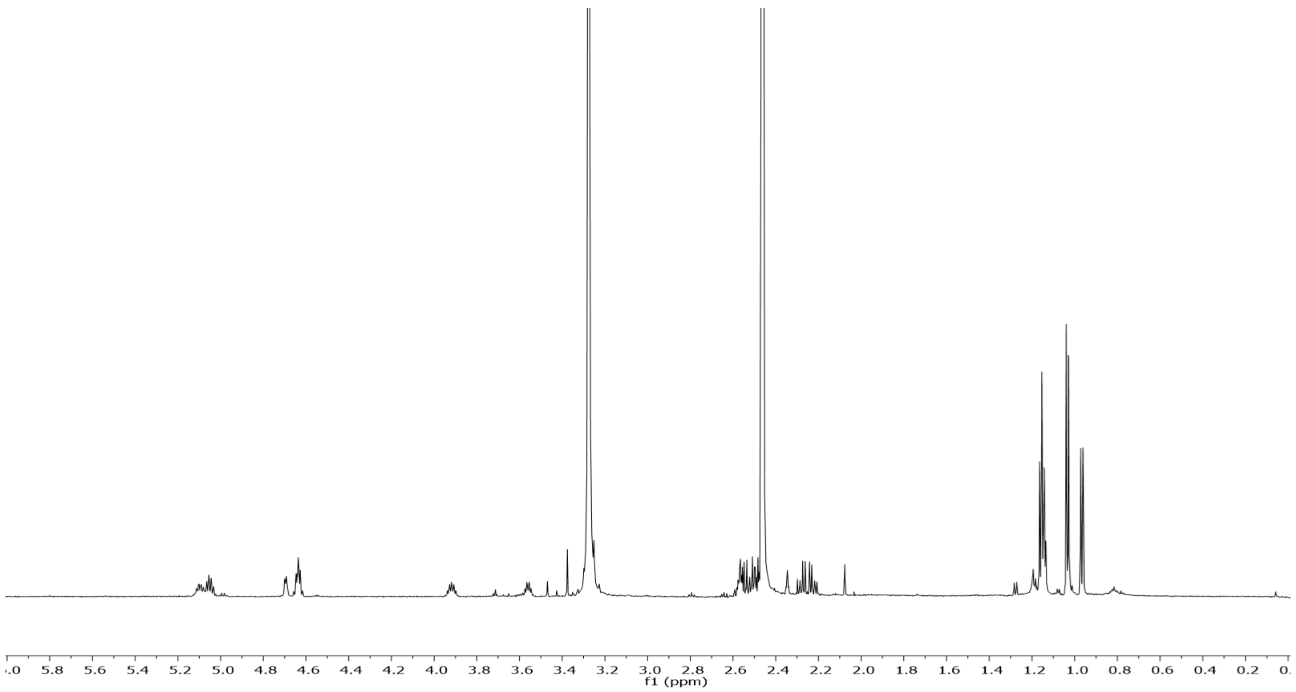

Supplementary Figure 11. COSY NMR spectrum (600 MHz) of polyhydroxybutyric acid derivatives in DMSO-d_6.

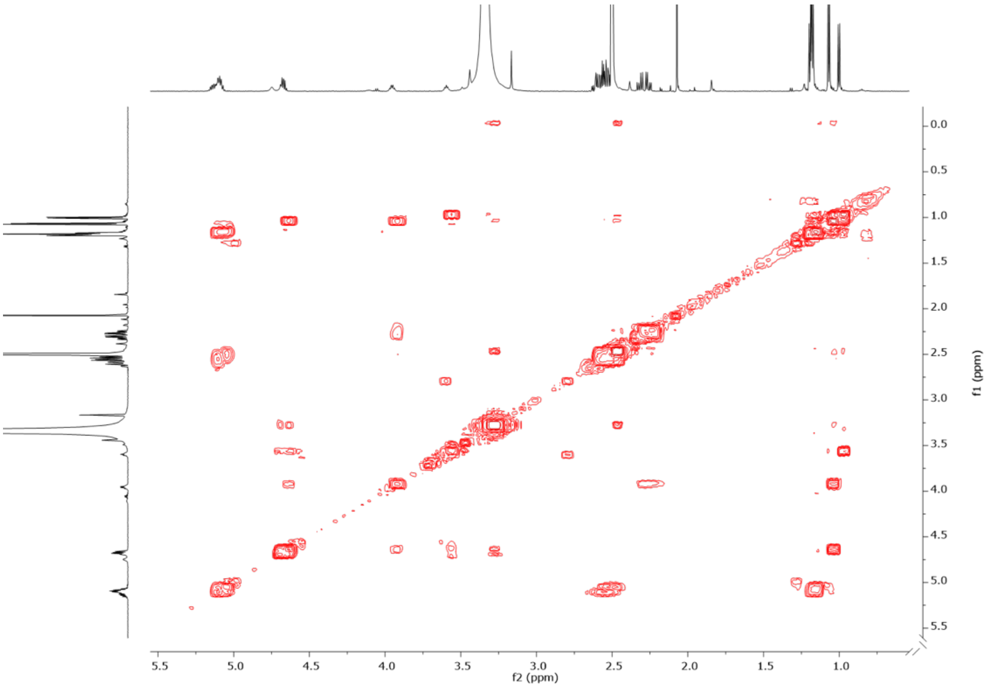

Supplementary Figure 11. HSQC NMR spectrum (600 MHz) of polyhydroxybutyric acid derivatives in DMSO-d_6.

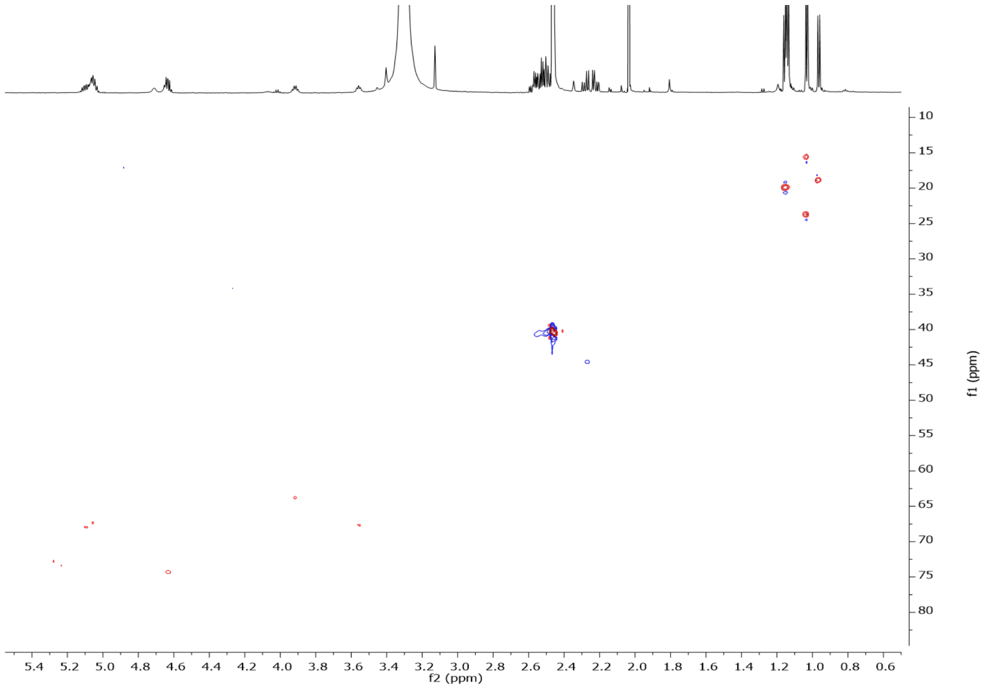

Supplementary Table 1. Taxon Composition List of *S. maritima* - Phylum

	Taxon name	Count	Proportion (%)
**1**	Proteobacteria	19,052	79.3701
**2**	Actinobacteria	3,749	15.6182
**3**	Bacteroidetes	484	2.0163
**4**	Firmicutes	404	1.6831
**5**	Cyanobacteria	221	0.9207
**6**	Rhodothermaeota	28	0.1166
**7**	Deinococcus-Thermus	17	0.0708
**8**	Verrucomicrobia	13	0.0542
**9**	Chloroflexi	11	0.0458
**10**	Acidobacteria	8	0.0333
**11**	Saccharibacteria_TM7	6	0.025
**12**	Planctomycetes	5	0.0208
**13**	Bacteria_uc	3	0.0125
**14**	Gemmatimonadetes	2	0.0083
**15**	Fibrobacteres	1	0.0042

Supplementary Table 2. Taxon Composition List of *S. maritima* - Class

	Taxon name	Count	Proportion (%)		Taxon name	Count	Proportion (%)
**1**	Gammaproteobacteria	16380	68.2386	**21**	Planctomycetia	5	0.0208
**2**	Actinobacteria_c	3714	15.4724	**22**	Anaerolineae	4	0.0167
**3**	Alphaproteobacteria	1529	6.3698	**23**	Balneolia	4	0.0167
**4**	Betaproteobacteria	1112	4.6326	**24**	MarineActino_c	4	0.0167
**5**	Bacilli	370	1.5414	**25**	PAC002280_c	4	0.0167
**6**	Cytophagia	257	1.0707	**26**	PAC002431_c	4	0.0167
**7**	Flavobacteria	184	0.7665	**27**	Thermoanaerobaculum_c	4	0.0167
**8**	Chroobacteria	174	0.7249	**28**	Bacteria_uc	3	0.0125
**9**	Hormogoneae	45	0.1875	**29**	Caldilineae	3	0.0125
**10**	Clostridia	26	0.1083	**30**	Erysipelotrichi	3	0.0125
**11**	Rhodothermia	24	0.1	**31**	Spartobacteria	3	0.0125
**12**	Sphingobacteriia	24	0.1	**32**	Verrucomicrobiae	3	0.0125
**13**	Deltaproteobacteria	22	0.0917	**33**	Chloroflexia	2	0.0083
**14**	Bacteroidia	19	0.0792	**34**	Kiritimatiellae	2	0.0083
**15**	Acidimicrobiia	17	0.0708	**35**	Longimicrobia	2	0.0083
**16**	Deinococci	17	0.0708	**36**	Thermomicrobia	2	0.0083
**17**	Nitriliruptoria	14	0.0583	**37**	PAC002560_c	2	0.0083
**18**	Oligoflexia	9	0.0375	**38**	Saccharimonas_c	2	0.0083
**19**	Negativicutes	5	0.0208	**39**	Chitinispirillia	1	0.0042
**20**	Opitutae	5	0.0208				

Supplementary Table 3. Taxon Composition List of *S. maritima* - Class

	Taxon name	Count	Proportion (%)		Taxon name	Count	Proportion (%)
**1**	Oceanospirillales	12260	51.0748	**35**	Methylococcales	5	0.0208
**2**	Pseudomonadales	3626	15.1058	**36**	Planctomycetales	5	0.0208
**3**	Kineosporiales	2023	8.4278	**37**	Puniceicoccales	5	0.0208
**4**	Micrococcales	1396	5.8157	**38**	Veillonellales	5	0.0208
**5**	Burkholderiales	1112	4.6326	**39**	Actinomarinales	4	0.0167
**6**	Sphingomonadales	1075	4.4784	**40**	Bacteriovoracales	4	0.0167
**7**	Bacillales	355	1.4789	**41**	Balneolales	4	0.0167
**8**	Cytophagales	257	1.0707	**42**	PAC000395_o	4	0.0167
**9**	Rhodobacterales	217	0.904	**43**	PAC002280_o	4	0.0167
**10**	Rhizobiales	206	0.8582	**44**	PAC002431_o	4	0.0167
**11**	Alteromonadales	202	0.8415	**45**	Thermoanaerobaculum_o	4	0.0167
**12**	Flavobacteriales	184	0.7665	**46**	Bacteria_uc	3	0.0125
**13**	Oscillatoriales	164	0.6832	**47**	Caldilineales	3	0.0125
**14**	Vibrionales	159	0.6624	**48**	Cellvibrionales	3	0.0125
**15**	Frankiales	148	0.6166	**49**	Chthoniobacterales	3	0.0125
**16**	Enterobacterales	111	0.4624	**50**	Erysipelotrichales	3	0.0125
**17**	Corynebacteriales	95	0.3958	**51**	Halanaerobiales	3	0.0125
**18**	Nostocales	45	0.1875	**52**	Oligoflexales	3	0.0125
**19**	Propionibacteriales	37	0.1541	**53**	Trueperales	3	0.0125
**20**	Rhodospirillales	29	0.1208	**54**	Verrucomicrobiales	3	0.0125
**21**	Rhodothermales	24	0.1	**55**	Arenicellales	2	0.0083
**22**	Clostridiales	23	0.0958	**56**	Bdellovibrionales	2	0.0083
**23**	Saprospirales	23	0.0958	**57**	Chromatiales	2	0.0083
**24**	Myxococcales	20	0.0833	**58**	Kallotenuales	2	0.0083
**25**	Bacteroidales	19	0.0792	**59**	Kiritimatiellales	2	0.0083
**26**	Acidimicrobiales	17	0.0708	**60**	Rickettsiales	2	0.0083
**27**	Lactobacillales	15	0.0625	**61**	DQ129389_o	2	0.0083
**28**	Deinococcales	14	0.0583	**62**	Desulfobulbaceae_o	2	0.0083
**29**	Pleurocapsales	10	0.0417	**63**	GU568020_o	2	0.0083
**30**	Xanthomonadales	10	0.0417	**64**	PAC000393_o	2	0.0083
**31**	Micromonosporales	9	0.0375	**65**	Saccharimonas_o	2	0.0083
**32**	Egibacterales	7	0.0292	**66**	Chitinispirillales	1	0.0042
**33**	Nitriliruptorales	7	0.0292	**67**	Sphingobacteriales	1	0.0042
**34**	Bifidobacteriales	5	0.0208	**68**	EF016806_o	1	0.0042

Supplementary Table 4. Taxon Composition List of *S. maritima* - Family

	Taxon name	Count	Proportion (%)		Taxon name	Count	Proportion (%)
**1**	Halomonadaceae	11945	49.7625	**41**	Phyllobacteriaceae	11	0.0458
**2**	Pseudomonadaceae	3620	15.0808	**42**	Enterobacteriaceae	10	0.0417
**3**	Kineosporiaceae	2023	8.4278	**43**	Micrococcaceae	10	0.0417
**4**	Microbacteriaceae	1357	5.6532	**44**	Xanthomonadaceae	10	0.0417
**5**	Oxalobacteraceae	1104	4.5992	**45**	Chroococcidiopsis_f	10	0.0417
**6**	Sphingomonadaceae	904	3.766	**46**	Lactobacillaceae	9	0.0375
**7**	Oceanospirillaceae	302	1.2581	**47**	Micromonosporaceae	9	0.0375
**8**	Paenibacillaceae	244	1.0165	**48**	Brevibacteriaceae	8	0.0333
**9**	Rhodobacteraceae	217	0.904	**49**	Saccharospirillaceae	8	0.0333
**10**	Hymenobacteraceae	198	0.8249	**50**	Reichenbachiella_f	8	0.0333
**11**	Flavobacteriaceae	177	0.7374	**51**	Acetobacteraceae	7	0.0292
**12**	Erythrobacteraceae	165	0.6874	**52**	Egibacteraceae	7	0.0292
**13**	Vibrionaceae	159	0.6624	**53**	Idiomarinaceae	7	0.0292
**14**	Pseudoalteromonadaceae	151	0.6291	**54**	Intrasporangiaceae	7	0.0292
**15**	Geodermatophilaceae	144	0.5999	**55**	Nitriliruptoraceae	7	0.0292
**16**	Symploca_f	98	0.4083	**56**	Marinobacter_f	7	0.0292
**17**	Nocardiaceae	95	0.3958	**57**	Marivirga_f	7	0.0292
**18**	Planococcaceae	83	0.3458	**58**	Carnobacteriaceae	6	0.025
**19**	Methylobacteriaceae	81	0.3374	**59**	Cytophagaceae	6	0.025
**20**	Erwiniaceae	66	0.275	**60**	Moraxellaceae	6	0.025
**21**	Devosia_f	49	0.2041	**61**	Planktothrix_f	6	0.025
**22**	Rivulariaceae	43	0.1791	**62**	Sphingomonadales_uc	6	0.025
**23**	Alteromonadaceae	37	0.1541	**63**	Bifidobacteriaceae	5	0.0208
**24**	Nocardioidaceae	37	0.1541	**64**	Planctomycetaceae	5	0.0208
**25**	Aurantimonadaceae	35	0.1458	**65**	Puniceicoccaceae	5	0.0208
**26**	Morganellaceae	35	0.1458	**66**	Veillonellaceae	5	0.0208
**27**	Prochlorotrichaceae	32	0.1333	**67**	Methylophaga_f	5	0.0208
**28**	Catalimonadaceae	31	0.1291	**68**	Alicyclobacillaceae	4	0.0167
**29**	CP003591_f	25	0.1041	**69**	Balneolaceae	4	0.0167
**30**	Rhizobiaceae	24	0.1	**70**	Comamonadaceae	4	0.0167
**31**	Bacillaceae	23	0.0958	**71**	Crocinitomicaceae	4	0.0167
**32**	Rhodospirillaceae	22	0.0917	**72**	Peredibacteraceae	4	0.0167
**33**	Rubricoccaceae	22	0.0917	**73**	AB478660_f	4	0.0167
**34**	Lewinellaceae	21	0.0875	**74**	AB533980_f	4	0.0167
**35**	Lachnospiraceae	17	0.0708	**75**	FM242290_f	4	0.0167
**36**	Bacteroidaceae	16	0.0667	**76**	HQ910322_f	4	0.0167
**37**	Acidimicrobiaceae	14	0.0583	**77**	PAC002137_f	4	0.0167
**38**	Deinococcaceae	14	0.0583	**78**	PAC002280_f	4	0.0167
**39**	Promicromonosporaceae	14	0.0583	**79**	PAC002431_f	4	0.0167
**40**	PAC000695_f	12	0.05	**80**	Ralstonia_f	4	0.0167

**81**	Thermoanaerobaculum	4	0.0167
**82**	Bacteria_uc	3	0.0125
**83**	Caldilineaceae	3	0.0125
**84**	Chthoniobacteraceae	3	0.0125
**85**	Clostridiaceae	3	0.0125
**86**	Cryomorphaceae	3	0.0125
**87**	Erysipelotrichaceae	3	0.0125
**88**	Halanaerobiaceae	3	0.0125
**89**	Hyphomicrobiaceae	3	0.0125
**90**	Oligoflexaceae	3	0.0125
**91**	Polyangiaceae	3	0.0125
**92**	Porphyromonadaceae	3	0.0125
**93**	Pseudanabaenaceae	3	0.0125
**94**	Trueperaceae	3	0.0125
**95**	Ilumatobacter_f	3	0.0125
**96**	Luteolibacter_f	3	0.0125
**97**	Anaplasmataceae	2	0.0083
**98**	Archangiaceae	2	0.0083
**99**	Arenicellaceae	2	0.0083
**100**	Cellvibrionaceae	2	0.0083
**101**	Chelatococcaceae	2	0.0083
**102**	Desulfobulbaceae	2	0.0083
**103**	Kallotenuaceae	2	0.0083
**104**	Kiritimatiellaceae	2	0.0083
**105**	Mooreiaceae	2	0.0083
**106**	Nostocaceae	2	0.0083
**107**	Sandaracinaceae	2	0.0083
**108**	Saprospiraceae	2	0.0083
**109**	CU925466_f	2	0.0083
**110**	DQ129389_f	2	0.0083
**111**	GU568020_f	2	0.0083
**112**	LRDG_f	2	0.0083
**113**	PAC000016_f	2	0.0083
**114**	PAC000616_f	2	0.0083
**115**	Bradyrhizobiaceae	1	0.0042
**116**	Chitinispirillaceae	1	0.0042
**117**	Chitinophagaceae	1	0.0042
**118**	Ectothiorhodospiraceae	1	0.0042
**119**	Exiguobacteriaceae	1	0.0042
**120**	Kangiellaceae	1	0.0042
**121**	Nannocystaceae	1	0.0042
**122**	Peptostreptococcaceae	1	0.0042
**123**	Ruminococcaceae	1	0.0042
**124**	Woeseiaceae	1	0.0042
**125**	AY913398_f	1	0.0042
**126**	EF016806_f	1	0.0042
**127**	Mogibacterium_f	1	0.0042
**128**	Pseudohongiella_f	1	0.0042
**129**	Saprospiraceae	2	0.0083
